# Notch and NF-κB: Coach and Players of Regulatory T-Cell Response in Cancer

**DOI:** 10.3389/fimmu.2018.02165

**Published:** 2018-10-11

**Authors:** Francesca Ferrandino, Paola Grazioli, Diana Bellavia, Antonio Francesco Campese, Isabella Screpanti, Maria Pia Felli

**Affiliations:** ^1^Department of Molecular Medicine, La Sapienza University, Rome, Italy; ^2^Department of Experimental Medicine, La Sapienza University, Rome, Italy

**Keywords:** Notch, NF-κB, regulatory T cells, Foxp3, cancer

## Abstract

The Notch signaling pathway plays multiple roles in driving T-cell fate decisions, proliferation, and aberrant growth. NF-κB is a cell-context key player interconnected with Notch signaling either in physiological or in pathological conditions. This review focuses on how the multilayered crosstalk between different Notches and NF-κB subunits may converge on Foxp3 gene regulation and orchestrate CD4^+^ regulatory T (Treg) cell function, particularly in a tumor microenvironment. Notably, Treg cells may play a pivotal role in the inhibition of antitumor immune responses, possibly promoting tumor growth. A future challenge is represented by further dissection of both Notch and NF-κB pathways and consequences of their intersection in tumor-associated Treg biology. This may shed light on the molecular mechanisms regulating Treg cell expansion and migration to peripheral lymphoid organs thought to facilitate tumor development and still to be explored. In so doing, new opportunities for combined and/or more selective therapeutic approaches to improve anticancer immunity may be found.

## Introduction

Regulatory T (Treg) cells are a heterogeneous population of T lymphocytes. Human and mouse Tregs act as gate-keepers of multiple immune reactions, suppressing unwanted immune responses such as autoimmunity, allergy, or transplant rejection (1–3). Treg cells (Tregs) are a first line of host-defense against infection, and prevent activation and expansion of autoreactive T cells. Infiltration of Tregs is associated with a decreased ratio of cytotoxic CD8^+^ T cells to Tregs (4), tumor progression (5), and poor prognosis in a number of cancers (6–8).

NF-κB transcription factors critically integrate the etiological mechanisms establishing inflammation as underlying malignancy (9), mainly orchestrating immune responses (10). NF-κB, triggered by multiple signaling pathways, in turn serves as a cell-intrinsic player in Treg development and function (Table 1). It also contributes as a multifaceted regulator being triggered and targeting gene expression regulation (17).

**Table 1:** Function of distinct NF-κB subunits in physiological T-reg activity and in cancer.

**NFκB Subunits**	**Physiological functions in Tregs**	**Pathological functions in Tregs**	**References**
**RelA/p65**	Development (nTregs). Acquisition/maintenance of mature Tregs identity and function.	Ablation results in autoimmune syndrome. Cooperatively with CSL up-regulates Foxp3 expression (nTregs).	(11–16)
**RelB**	Not-intrinsically required for development or suppressive function. Development in intact thymic medulla (nTregs) by a Treg-extrinsic mechanism. Peripheral Treg homeostasis under p100 control.	Loss induces systemic autoimmunity and expansion of Foxp3^+^ Tregs (Treg-extrinsic mechanism). Mediates SDF1/CXCR4 axis at the tumor site (Treg-extrinsic mechanism).	(11, 17–19)
**c-Rel**	Development (nTregs). Maintenance of numbers and identity (nTregs). Homeostatic expansion (iTregs).	Inhibition of antitumor responses. Migration to inflamed tissues and tumors (aTreg). Maintenance of numbers and identity at the tumor site (aTreg). Loss induces mild autoimmunity.	(11, 13, 20–22

*Treg identity is (Gitr^+^CD25^+^Foxp3^+^); nTregs, natural Tregs; iTregs, induced Tregs; aTregs, activated Tregs; CSL-the transcriptional repressor CBF1/suppressor of hairless/Lag-1 (or the human homolog RBPJk-recombining binding protein suppressor of hairless); Stromal cell-derived growth factor 1 (SDF1)/CXCR4; p100 subunit encoded by the NF-kB2 gene*.

Natural Treg (nTreg) arising in the thymus very early after birth and induced Treg (iTreg) in the periphery are both influenced by Notch signaling, notably in a cell context-dependent pathway (23). Notch signaling promotes the generation and function of nTreg, but its inhibition enhances Treg functions and protects mice from graft-vs.-host disease (24, 25). In intact thymic medulla, Tregs during their development require RelB-dependent functions of medullary thymic epithelial cells, which also provide co-stimulatory molecules and MHC class I/II (18).

Many authors have contributed to unveiling the key features of both Notch and NF-κB pathways in Treg biology, also in the context of a tumor. Here we will focus on some important clues related to the functional plasticity of the two signaling pathways, and to their interplay still unexplored in the regulation of Treg expansion and function in cancer.

## The NF-κB team in treg biology

The mammalian NF-κB family is composed of five members, p65 (RelA), RelB, c-Rel, p105/p50, and p100/p52, which originate a collection of homodimers and heterodimers (26), that are tightly controlled and sequestered into the cytoplasm by IκB, NF-κB inhibitory proteins.

NF-κB activation occurs through two pathways depending on the components of the IκB kinase (IKK) complex: the canonical heterotrimer IKKα/IKKβ/IKKγ and the alternative IKKα/IKKα homodimer (17), which is required for the homeostasis of Tregs and for the expansion of both regulatory and effector CD4^+^ T cells (27). Next, IKKβ-dependent serine-phosphorylation and ubiquitin-dependent degradation of IκBα initiate canonical NF-κB dimer (p50/p65) activation and nuclear entry (17). Notably, p65 and c-Rel (encoded by *Rela* and *Rel*, respectively) drive the acquisition/maintenance of Treg identity (Gitr^+^CD25^+^Foxp3^+^) and function (11). In contrast, the conditional deletion of RelB in Foxp3^+^ Tregs does not alter the number and function of this subset, even though the germline deletion of RelB induces autoimmunity and an expansion of Foxp3^+^ Tregs (Table 1), mainly due to T cell-extrinsic mechanisms (19).

In the context of T cells, multiple extracellular signaling cascades including Notch (28, 29) can converge on the canonical NF-κB pathway. This may also be triggered by the pre-T-cell receptor (pre-TCR) (30) whose functional cooperation with constitutive Notch3 expression is involved in the pathogenesis of a Notch3-induced T-cell acute lymphoblastic leukemia (T-ALL) (31) characterized by a wide CD4^+^CD25^+^Treg expansion (32, 33).

Regarding the alternative signaling pathway, NF-κB-induced kinase (NIK) phosphorylates to activate IKKα, which promotes p100 (encoded by *NF-*κ*B2*) precursor protein processing. This then generates the main “alternative” complex p52/RelB that crucially controls lymphoid organogenesis and cell migration (34).

Interestingly, Murray et al. genetically manipulated the NIK expression in mice and demonstrated that the NIK deletion in T cells specifically impairs the maintenance of peripheral Foxp3^+^ Tregs, thus suggesting a Tregs intrinsic function for the noncanonical pathway (35). Alternatively, the lineage-specific constitutive activation of NIK in Treg cells induces an alteration of their functions and gene signature (Gitr^+^CD25^+^Foxp3^+^), leading to the development of an autoimmune syndrome (36).

**Figure 1:**
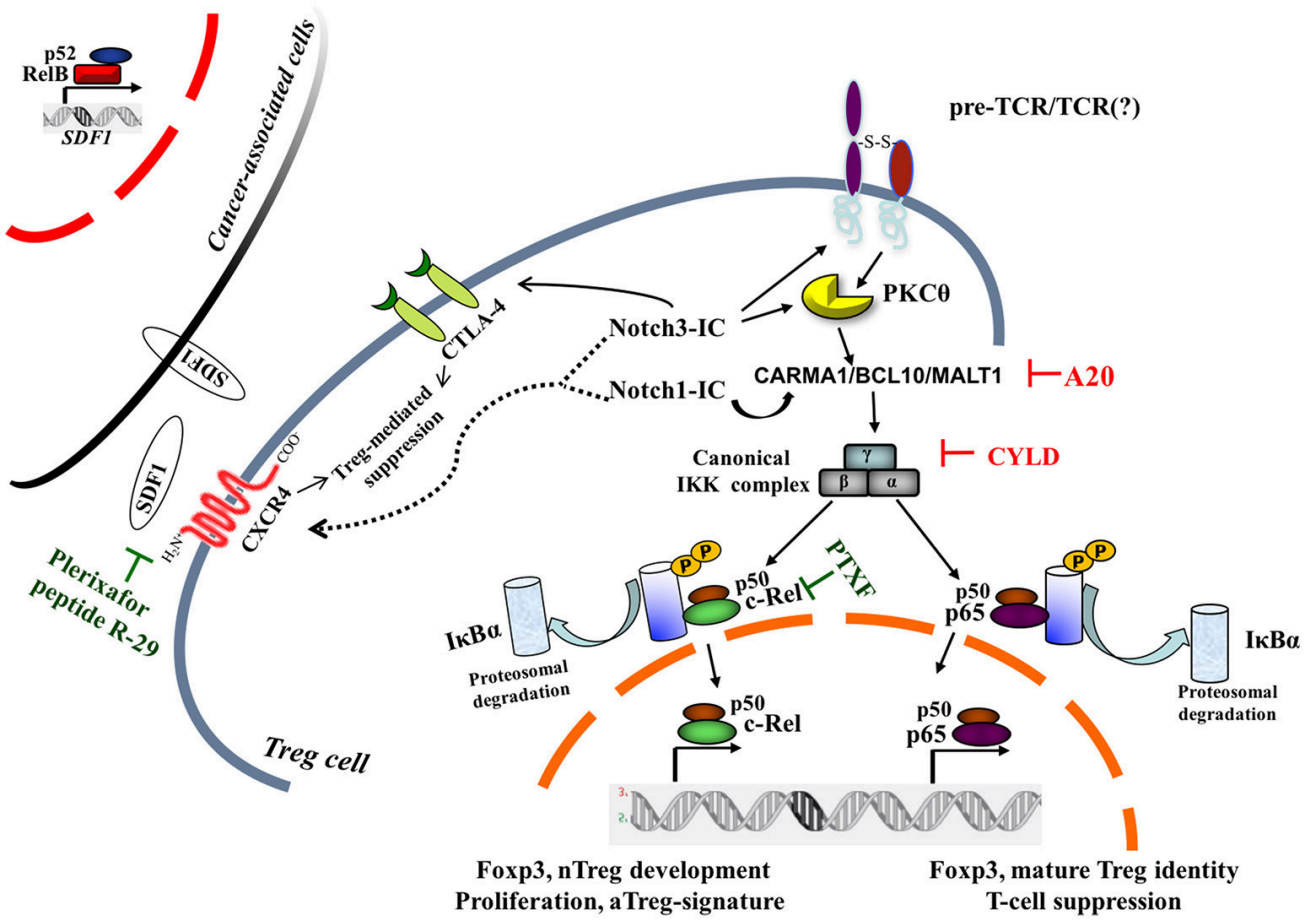
Canonical NF-κB pathway is central to intrinsic Notch1- and Notch3-modulated Treg cell function within tumor microenvironment. Two NF-κB negative regulators, A20 and CYLD, on removal of nonproteolytic K63-linked polyubiquitin chains from signaling molecules, interfere with the preTCR/TCR pathway, leading to NF-κB activation. For a pharmacological approach, pentoxifylline (PTXF) that selectively degrades c-Rel is indicated, as well as inhibitors of Treg-mediated suppression activity by CXCR4 antagonists, such as plerixafor (AMD3100) or peptide R-29. The dotted line refers to hypothetical Notch1- and/or Notch3-induced CXCR4 modulation in Treg cells, whereas the black curved-line indicates the Notch3-enhanced CTLA4 expression in N3-ICtg Tregs (36). Cancer-associated cells once activated in a tumor microenvironment can express many proinflammatory genes, including stromal cell-derived factor 1 (SDF1), the cognate ligand of CXCR4, partly in an NF-κB-dependent manner (23). pTα-chain (preTCR) and T-cell receptor (TCR); IκBα, inhibitor of NF-κBα.

In mature T cells, upon the engagement of the TCR/CD28 complex, PKCθ and the CARMA1/BCL10/MALT1 (CBM) protein complex are recruited to finally induce NF-κB activation (37) (Figure 1). Mutations of TCR signalosome (CBM-PKCθ-IKKβ) components selectively impact nTreg biology, whereas conventional T-cell development seems to be less affected (38–41). Notably, Notch1 can also initiate NF-κB activation via cytosolic interactions with T-cell signalosome components (42). PKCθ-selective transport to lipid rafts within the immunological synapse (43) will recruit IKK to the CBM and trigger IKK activation; this pathway is negatively regulated by the deubiquitinase CYLD. *CYLD*-deficient mice display constitutive NF-κB activation in thymocytes and peripheral T cells. The Treg frequency is enhanced although Tregs are less functional than the *wild-type* counterparts (44). Recently, it was demonstrated that another negative regulator of NF-κB—ubiquitin-editing enzyme A20—restricts nTreg development; however, A20-/- Tregs are completely functional *in vivo* (45). Interestingly, while A20 terminates NF-κB signaling, CYLD prevents spontaneous NF-κB activation. Notch3 overexpression in combination with the pTα/preTCR function increases Lck-dependent PKCθ translocation to the cell membrane, triggering PKCθ/IKKβ-axis hyperactivation (46). Intriguingly, PKCθ and CYLD are antagonistic partners in the NF-κB activation in T cells (47). However, PKCθ is involved in Treg cell differentiation *in vivo*, but it is dispensable for Treg-mediated suppression (48); therefore, the balance between the positive (PKCθ) and/or negative (CYLD, A20) regulators of NF-κB may govern the generation and function of Tregs (Figure 1).

Post-translational modifications can also fine-tune the transcriptional activity of nuclear NF-κB to modulate its interaction with coactivators, corepressor, IκB proteins, and the binding to heterologous transcription factors (enhanceosomes), thus shaping NF-κB-dependent gene programs (10).

In particular, the phosphorylation of serine 276 and additional residues are critical for CBM recruitment and the transcriptional activity of p65 (10). Notably, IKKβ-mediated phosphorylation of p65/serine 536 has been shown to require PI(3)K-Akt activity, an emerging node for crosstalk between NF-κB and PI(3)K-Akt pathways, whose balance is important in promoting selection into the Treg-cell lineage (49) (and references therein). Interestingly, the phosphorylation of the p65/serine 536 residue is strongly promoted in Notch3-induced T-ALL (50).

## NF-κB: a forward player of tregs activity in cancer

The infiltration of Tregs into various tumor tissues promotes tumor progression by limiting the antitumor immune response and the supporting tumor immune evasion (4, 6, 51). Tregs exert these functions, as a combined result of efficient migration into the tumor site, local expansion of specialized subsets, and *de novo* generation within the tumor, all of which are still poorly unveiled. A highly immunosuppressive Treg subtype, expressing tumor-necrosis-factor-receptor 2 with activated NF-κB/p65 has been abundantly recognized in human ovarian cancers (52). In human hepatocellular carcinoma, the decreased survival rate was associated to a higher level of peripheral blood Tregs; similar observations have been reported in chronic lymphocytic leukemia (CLL) patients (53). However, controversial is the role of high Treg infiltration as a prognostic parameter in colorectal cancer (54, 55).

In mice, the resting Tregs (rTregs) resident in lymphoid tissues prevent lymphoproliferative disease and autoimmunity, and are maintained by the Foxo1-activated transcription function (5, 56). On the contrary, the “effector-memory like” activated Treg subset (aTreg) migrates to the inflamed tissues and tumors and potently inhibits antitumor responses (20–22), essentially associated to the c-Rel function (Table 1) (57).

Tregs typically suppress T-cell proliferation and cytokine production in target CD4^+^ T cells. This inhibition is achieved by reducing nuclear NF-κB/p65 accumulation (58).

Reversibly, in mice, the inhibition of the canonical NF-κB pathway by the “super repressor” IkBSR-enforced expression or the IKKβ loss impairs Tregs development (39), whereas the genetic ablation of canonical NF-κB proteins (c-Rel) profoundly reduces the numbers of CD4^+^Foxp3^+^ Tregs in the neonatal and adult thymus and in peripheral lymphoid organs (59, 60).

During the development of nTregs inside the thymus, both the nuclear localization and activity of c-Rel and RelA have been described in the transition from CD4^+^CD8^+^ (DP) to Treg precursors generation (CD25^hi^Gitr^hi^Foxp3^−^CD4^+^) (12). Elegant studies by Gosh et al. demonstrated that canonical NF-κB members have unique but partially redundant roles in Treg biology, with c-Rel being critical for thymic Treg development and p65 essential for mature Treg identity and maintenance of immune tolerance (11, 13). Indeed, c-Rel loss decreases the number of nTregs and the expression of Treg signature genes (Gitr, CD25, Foxp3) involved in the maintenance of Treg identity (11), whereas mice harboring the p65 ablation in Tregs develop a lethal autoimmune syndrome. However, in the tumor context, the same group demonstrated that melanoma growth is drastically reduced in mice lacking c-Rel, but not p65, in Tregs. Strikingly, the selective degradation of c-Rel, by pentoxifylline, delays tumor growth by altering Treg function and identity (Figure 1) and potentiates anti-PD-1/PD-L1 therapy (57). Therefore, c-Rel modulates activated Treg functions.

As for the alternative pathway of NF-κB activation, conditional NIK overexpression in T cells expands both the Treg and the activated conventional T-cell subsets; however, Tregs are largely nonfunctional allowing conventional T cells (Tconvs) to escape suppression, thus inducing a lethal inflammation in mice (61). Recently, it was demonstrated that the conditional deletion of the p100 gene in Tregs causes a massive inflammation due to the impaired suppressive function of *NF-*κ*B2/p100*-deficient Tregs, revealing an increased nuclear translocation of RelB responsible for the accumulation of Tregs *in vivo* (Table 1) (62). To date, it remains to be elucidated if the modulation of the alternative pathway of NF-κB leads to similar effects in cancer.

## Notch and notch/NF-κB signaling crosstalk as a playmaker of tregs in cancer

The Notch signaling pathway has been repeatedly associated with different aspects of Treg biology (63), but the potential effect of Notch and its privileged crosstalk with the canonical NF-κB pathway on Treg behavior in cancer is still poorly understood.

Recent evidence has demonstrated that elevated Notch signaling positively modulates peripheral Treg numbers and function in different tumor microenvironments, as demonstrated in the head and neck squamous-cell carcinoma (HNSCC) (64) and even associated to the pathological aggressiveness in human pancreatic (intraductal papillary mucinous) tumors (65).

The study reported in (65) demonstrated that the enhancement of Tregs in the peripheral blood samples of patients affected by a pancreatic tumor fairly correlated to the higher expression of Notch1 and Notch2, while the elevated expression of the Notch/ligand, Jagged1, was related to recurrence (65). Accordingly, in HNSCC, Notch inhibition reduced Tregs, myeloid-derived suppressor cells, tumor-associated macrophages, and the expression of immune checkpoint molecules in the circulation and in the tumor (64). More selectively, Notch1 has been associated to Tregs infiltration in a subset of human breast luminal tumors (66).

Life-and-death decisions in Tregs are influenced by Notch subcellular localization. In fact, when in cytosol, Notch1 protects Tregs from apoptosis induced by cytokine withdrawal (67). The microenvironment can even modulate Notch localization in Treg. In a nutrient-limiting condition, sirtuin 1 stabilizes the Notch intracellular domain (N-ICD) proximal to the plasma membrane and promotes the survival and function of Tregs (68). Therefore, tumor microenvironmental changes may tune noncanonical Notch1 signaling in Treg activities.

Canonical and noncanonical Notch signaling play key roles, often in conjunction with NF-κB, in the Treg-dependent immunological response to the cancer (69, 70). Upon ligand binding, the Notch extracellular subunit is released and trans-endocytosed by the ligand-expressing cell, and this probably activates the genetic programs in stromal cells apt to modulate either thymocyte development (i.e., oxp3^+^ nTregs) or the tumor microenvironment. In the receptor-bearing cell, three subsequent proteolytic cuts release N-ICD. Subsequently, N-ICD translocates to the nucleus and interacts with the DNA-binding CSL/RBP-Jk factor (71). This drives N-ICD to the target gene promoter, where it recruits mastermind-like (MAML) and additional coactivators, finally driving target gene expression in a wide spectrum of tissues or in a tissue-restricted way. In fact, Notch1-IC can directly bind on RelB and p52 promoters potentially recruiting the MAML1/CSL complex (72).

The crosstalk of Notch with NF-κB in T-cell development (73) as well as in Notch-induced T-cell leukemogenesis has been extensively reported by our group that generated a Notch3 transgenic mice (N3-ICtg) (28, 31, 46, 50). Intriguingly, this murine model is also characterized by enhanced CD4^+^CD25^+^CTLA4^+^ Tregs generation (32), suggesting that Notch/NF-κB crosstalk may modulate Treg behavior in cancer.

Notch and NF-κB, both activated in several cancer scenarios, display a multilayered crosstalk. Directly, Notch1 modulates the expression of NF-κB subunits in T-cell leukemia (74) or, indirectly it binds to NF-κB subunits to modulate the transcriptional outcomes in a specific context and cell type (75). Upstream, Notch1 may associate with IKKα, activating NF-κB in cervical cancer cells (76). Unlike Notch1, neither the upregulation of NF-κB subunit expression by Notch3 hyperactivation nor a direct binding between these two partners has been reported so far.

In a different context, the noncanonical Notch1 signaling, independently from RBP-jk, but likely through NF-κB, regulates the activation and proliferation of CD4^+^ T cells and the differentiation of iTreg lineage (77).

Conversely, NF-κB can trigger Notch ligands, Jagged1 (78) and Jagged2 (79), both increasing Tregs generation (80) and recently found upregulated in hair-follicle-resident Tregs that form an immune-privileged niche for stem cell biology. Few papers correlated the two ligands to CD4^+^CD25^+^Foxp3^+^ expansion in inflammation (81) and in pancreatic tumors (65), thus suggesting Jagged as an important area of investigation in cancer-associated Tregs. Already in clinical trials, therapeutic antibodies inhibiting ligand/receptor interactions would be informative and a valuable drug in cross-signaling between Tregs, stroma, and Notch-expressing cancer cells.

To exploit their effects on tumor progression, Tregs need to migrate into tumor sites. In this context, it has been recently demonstrated that Tregs homing to the bone marrow is CXCR4-mediated (29, 30, 82) (and references therein). In fact, CXCR4 is critical for Notch3-enhanced T-cell leukemia propagation (83) and in the maintenance in the bone marrow of Notch1-induced T-ALL cells (84) that are characterized by the constitutive activation of NF-κB (28, 50, 85). In the neoplastic context, CXCR4 expression has been linked to NF-κB signaling activation (86). Additionally, CXCR4 antagonism (AMD3100) (Figure 1) reverts the suppressive activity of activated Tregs (CTLA4^+^/CXCR4^+^/PD-1^+^/ICOS^+^) in renal cancer (87) or reprograms Tregs in human mesothelioma (88). Therefore, we can suggest a Notch/CXCR4 connection in potentiating Treg activities, resulting in a protective immunosuppressive environment for T-ALL cells.

## Factors playing on Foxp3 promoter

In the primary CD4^+^ environment, Foxp3 expression marks the commitment to CD4^+^CD25^+^Foxp3^+^ Tregs (89) and is required for suppressive activity and transcriptional repression (90). Foxp3 regulates gene expression either by associating with other nuclear factors (91, 92) or antagonizing the NF-AT function by directly competing for DNA binding to consensus forkhead binding sites adjacent to NF-AT (93). Furthermore, Foxp3 over-expression may indirectly impair the translocation of NF-κB into the nucleus by increasing IκB-α stability, thus preventing p65 nuclear entry (94).

On the other side, multiple signaling pathways converge on Foxp3 modulation (93, 95, 96). Three different groups highlighted the central role of the canonical c-Rel transcription factor in Foxp3 gene expression (59, 97, 98). Indeed, c-Rel cooperatively with NF-AT binds to the Foxp3 promoter to form a Foxp3-specific enhanceosome (c-Rel/p65/Smad3/NFATc2/CREB) and recruits chromatin-modifying complexes to the regulatory sequences shortly before the appearance of Foxp3^+^ thymocytes in the CD4^+^ T-cell compartment (98).

Dispensable for nTregs development, TGFβ signaling critically regulates peripheral Treg (iTreg) number and functionality and induces Foxp3 expression (99, 100), whereas c-Rel is required only for the optimal homeostatic expansion of iTregs. Indeed, CD28 co-stimulus preferentially triggers RelA to activate Foxp3, at least in human iTregs (101).

Finally, the Foxp3 promoter behaves as an integration site between canonical NF-κB and different signaling pathways (102) that could cooperatively or antagonistically influence Tregs behavior in tumor microenvironments.

## Notch3 and NF-κB kick-starters in Foxp3 promoter activation

Several papers have highlighted the multiple roles served by Notch and/or NF-κB pathways in regulating Foxp3 gene expression (63, 102).

Our group revealed the importance of Notch signaling activation in driving Tregs generation and functions by demonstrating the higher levels of Notch3 in CD4^+^CD25^+^ with respect to CD4^+^CD25^−^ T cells (32). Moreover, we also showed that Notch3/preTCR cooperation increases both Foxp3-expressing Treg population numbers and Foxp3 expression, as well as enhances *in vivo* activity of nTregs (33).

Other groups demonstrated that Notch1, together with TGFβ, regulates Foxp3 expression and the maintenance of peripheral iTregs (103).

Notch and NF-κB can regulate multiple steps in different T-cell subsets, but neither the mere absence of NF-κB (104) nor the Notch deregulation alone (14) impair numbers and frequencies of the total CD4^+^ T-cell compartment.

However, we demonstrated that the Notch3 hyperactivation in the N3-ICtg murine model of T-ALL requires PKCθ signals to upregulate Foxp3 core-promoter and to regulate Foxp3^+^ T-cell generation and suppressive function (14). Therefore, Notch3 and PKCθ converge on the hyperactivation of the canonical NF-κB pathway that rules over the developmental aspects and the activity of Tregs in the tumor microenvironment (11). Interestingly, constitutive NF-κB activation in two different *CYLD*-deficient murine models enhances Foxp3 expression and increases the total amount of Foxp3^+^ Tregs in the thymus and lymph nodes (44, 105).

Standing the PKCθ/CYLD antagonism, we can hypothesize that the PKCθ hyperactivation observed in N3-ICtg thymocytes may suppress the CYLD function, thus further sustaining NF-κB activation, in agreement with Notch/Hes1-induced CYLD repression and reduced expression of this IKK negative regulator in primary T-cell leukemia (85).

The enhanced generation of Tregs in the thymus is strictly linked to Foxp3 induced by NF-κB family partners sequentially activated. This picture can be further complicated by the arrival of Notch3 signals that can recruit on the Foxp3 promoter a new complex binding the p65/CSL-nested site close to the transcription start site of the Foxp3 promoter (Table 1) (14). More importantly, we can suggest that this Notch/p65 cooperation can be active also in the regulation of Foxp3 signaling in cancer cells (106), as recently described in thyroid cancer and T-ALL (15, 16).

Compendiously, the crosstalk between hyperactive Notch3 and canonical NF-κB pathways upregulates Foxp3 expression, thus enhancing the suppressive function of Tregs against protective antitumor immune responses in tumor microenvironments.

## Conclusion and perspectives

The increased number of Tregs within peripheral blood, lymphoid tissue, and the tumor microenvironment is frequently associated with poor prognosis in several cancers (i.e., ovarian, gastric, breast, and renal cancer). Specifically targeting the Treg compartment while sparing other T-cell populations, which may be useful in tumor immune response, is difficult. Many chemotherapeutic agents (cytostatic drugs) impinge on the increased proliferative rate of Tregs in cancer patients but still with a limited selectivity (107). Further research is required to develop Treg-specific depletion strategies to favor immune response against malignant cells.

In this mini-review, we discussed the intricate network that governs Foxp3 transcription and Treg generation and function, particularly emphasizing the role played either by Notch or by NF-κB signaling, or newly, by their convergence in T-cell leukemia. The multilayered Notch/NF-κB interplay may suggest new issues to be targeted in “cell-intrinsic” mechanisms driving Foxp3-mediated activities of Tregs. In the future, we need to explore the relative role of crosstalk between specific Notch receptors and NF-κB subunits within the subsets of tumor-associated Tregs and importantly their interplay with cancer and microenviromental cells. Therefore, selective γ-secretase-inhibitors or therapeutic antibodies with Notch-specific affinity may suppress the selected Tregs, thus contributing to combined chemotherapy. Innovative cancer immunotherapies target Treg surface receptor and effector T cells, possibly impinging on the abnormal NF-κB-mediated Tregs activity (52). Therapeutic Notch modulation could enhance the efficacy of immunotherapy firstly acting as the immune modulator by reinforcing the T cells' antitumor effector function and secondly behaving as NF-κB partner by impinging on the intrinsic mechanisms of Tregs and cancer-associated cells. Therefore, elucidating the role of both pathways could be a valuable tool to design specific treatment plans aimed to decrease drug dosage and toxicity. Notch and NF-κB profiles may contribute to identify patients and tumors likely to respond to immunotherapy and to provide a new alternative approach to nonresponders. Promising therapies implied that Notch modulation (anti-Jagged1/2) combined with novel immune checkpoint blockade therapies (108).

Still unresolved is the wide partnership of ubiquitous Notch and NF-κB subunits in regulating Foxp3 and Tregs transcriptional programs, and even more the reason why the hyperactivation of either Notch or NF-κB signaling pathway is insufficient to generate fully mature Tregs. The knowledge of specific NF-κB subunits that are upregulated in cancer-associated Tregs will have a clear impact in the development of selective immunomodulatory therapeutics that target NF-κB, by performing a subunit-specific inhibition in Tregs, as suggested by Pentoxyphylline, an FDA-approved drug.

Treg targeting approaches may also include a strategy to interfere with microenvironmental signals, mostly represented by the chemokine receptor/ligand system, as CXCR4-mediated Treg homes to the tumor. It will be insightful also to decipher the cross-signaling in regulating the Foxp3 expression in different Notch-governed cell contexts such as in T-ALL cells (16, 109). The final aim of all these studies would be to define innovative anticancer therapeutic approaches with genetically modified Tregs (110) to treat cancer.

## Author contributions

FF researched the literature and wrote the initial draft of the manuscript. PG performed the literature review and helped in editing the table. AFC and IS critically revised the manuscript. DB performed the literature review and helped in editing the figure. MPF wrote the manuscript and edited the figure and table.

### Conflict of interest statement

The authors declare that the research was conducted in the absence of any commercial or financial relationships that could be construed as a potential conflict of interest.
